# HCMV-pUS2 Disrupts cGAS-STING Signaling through LMAN2L Degradation

**DOI:** 10.1371/journal.ppat.1014246

**Published:** 2026-05-18

**Authors:** Yue-Peng Zhou, Yong-Xuan Yao, Jin-Peng Wu, Yu-Ting Pan, Wen-Bo Zeng, Jin-Yan Sun, Yue Zhao, Han Cheng, Min-Hua Luo, Bo Yang

**Affiliations:** 1 State Key Laboratory of Virology and Biosafety, Wuhan Institute of Virology, Chinese Academy of Sciences, Wuhan, China; 2 University of Chinese Academy of Sciences, Beijing, China; 3 State Key Laboratory of Magnetic Resonance Spectroscopy and Imaging, National Center for Magnetic Resonance in Wuhan, Innovation Academy for Precision Measurement Science and Technology, Chinese Academy of Sciences, Wuhan, China; 4 Department of Pediatric Surgery, Guangdong Provincial Key Laboratory of Research in Structural Birth Defect Disease, Guangdong Provincial Children’s Medical Research Center, Guangzhou Institute of Pediatrics, Guangzhou Women and Children’s Medical Center, Guangzhou Medical University, Guangzhou, China; 5 Shanghai Public Health Clinical Center, Fudan University, Shanghai, China; State University of New York Upstate Medical University, UNITED STATES OF AMERICA

## Abstract

Human cytomegalovirus (HCMV) has evolved diverse strategies for immune evasion. In this study, we identified HCMV-pUS2 as an indirect antagonist of the cGAS-STING pathway by promoting the degradation of lectin mannose-binding 2-like protein (LMAN2L), an unrecognized host factor involved in STING pathway. First, we discovered that HCMV, but not other DNA viruses such as HSV-1 and VACV, induces proteasomal degradation of LMAN2L during the immediate-early stage of infection. We then demonstrated that HCMV-pUS2 mediates LMAN2L degradation by recruiting the host E3 ubiquitin ligase RNF139 and E2 ubiquitin-conjugating enzyme UBE2G2, directing LMAN2L to the endoplasmic reticulum (ER)-associated protein degradation (ERAD) pathway. LMAN2L knockout diminishes HCMV-induced expression of type I interferons and interferon-stimulated genes. Furthermore, LMAN2L co-localizes and interacts with STING. Though it does not affect STING dimerization or TBK1 recruitment, it is essential for STING translocation from the ER to the Golgi. Our findings uncover LMAN2L as a novel host regulator of the STING pathway and identify pUS2-mediated ERAD as a previously unrecognized viral immune evasion strategy.

## Author summary

HCMV employs sophisticated strategies to target the cGAS-STING pathway for immune evasion. This study identifies HCMV-pUS2 as a novel negative regulator of STING signaling. We elucidate the details of pUS2-mediated LMAN2L degradation and characterize LMAN2L as a previously unrecognized STING-interacting protein that facilitates STING translocation from the ER to the Golgi following activation. These findings reveal a new mechanism of HCMV immune evasion and provide valuable insights into the molecular basis of STING trafficking, with implications for anti-HCMV therapy development and innate immunity research.

## Introduction

Human cytomegalovirus (HCMV) is a ubiquitous pathogen that has evolved to coexist with human hosts, with an estimated 60–90% of adults being seropositive worldwide [1]. While the infection is typically asymptomatic in immunocompetent individuals, HCMV establishes latency within the host and persists for life [2]. HCMV infection is a significant risk factor in bone marrow transplant failures and a leading cause of birth defects [3,4]. Approximately 37–60% of patients receiving allogeneic hematopoietic stem cell transplantation suffer from HCMV infection, even when treated with antivirals [5,6]. In addition, congenital HCMV infection affects about 0.64% of newborns [7], with 5–10% of these infants displaying symptoms at birth, including neurological impairments, developmental delays, epilepsy, spastic quadriparesis and sensorineural hearing loss [7–9].

To counter HCMV, the hosts utilize pathogen recognition receptors to detect viral elements and trigger the production of type I interferon (IFN-I), which subsequently activate the expression of interferon-stimulated genes (ISGs) [10]. The cyclic guanosine monophosphate-adenosine monophosphate (cGAMP) synthase (cGAS) is a key cytosolic DNA sensor that detects HCMV in multiple human cell types [11,12]. Upon recognizing double-stranded DNA (dsDNA), cGAS uses ATP and GTP as substrates to generate the second messenger cGAMP [13], which then activates the endoplasmic reticulum (ER)-localized adaptor protein the stimulator of interferon genes (STING, also known as MITA, ERIS, MPYS, or TMEM173) [14–17]. STING oligomerizes and translocates to the Golgi apparatus, where TANK-binding kinase 1 (TBK1) is recruited to the STING signalsome to phosphorylate interferon regulatory factor 3 (IRF3). Once activated, IRF3 relocates to the nucleus and triggers the expression of type I IFNs and ISGs [18], thereby restricting HCMV replication.

During co-evolution with human hosts, HCMV has developed various mechanisms to modulate the cGAS-STING signaling pathway, facilitating efficient infection, as well as establishing and/or maintaining latency. Ten HCMV-encoded proteins (pUL31, pUL35, pUL42, pUL48, pUL82, pUL83, pUL94, pUL122, pUL138, and pUS9) have been identified as inhibitors of the cGAS-STING pathway, effectively undermining host antiviral responses [19–28]. These proteins belong to different temporal classes and act at various stages of HCMV infection to modulate cGAS-STING activation. Most of their inhibitory effects result from direct interactions with components of the pathway, ultimately leading to the suppression of downstream transcription of IFNs and ISGs.

A previous multiplexed screen systematically identified viral-targeted host proteins during early HCMV infection [29]. Among these, HCMV restriction factors such as DAXX, Sp100, and MORC3 were found to be degraded to evade intrinsic immunity, consistent with previous studies [30–33]. LMAN2L was recently shown to be degraded by pUS2-mediated ER-associated degradation (ERAD) *via* the E3 ubiquitin-protein ligase RNF139 (also known as TRC8), resulting in impaired trafficking of cell-surface integrins. However, the biological role of this downregulation remained unclear. In this study, we provide a comprehensive characterization of pUS2-mediated LMAN2L degradation, revealing recruitment of UBE2G2 and RNF139 to facilitate K48-linked polyubiquitination of LMAN2L. Importantly, we uncover that LMAN2L facilitates STING translocation to the Golgi, a process targeted by pUS2 through ERAD-mediated LMAN2L degradation. These findings establish pUS2 exploitation of ER quality control as a novel mechanism of STING signaling subversion by HCMV.

## Results

### HCMV infection decreases LMAN2L protein level at the immediate-early stage

**Fig 1:**
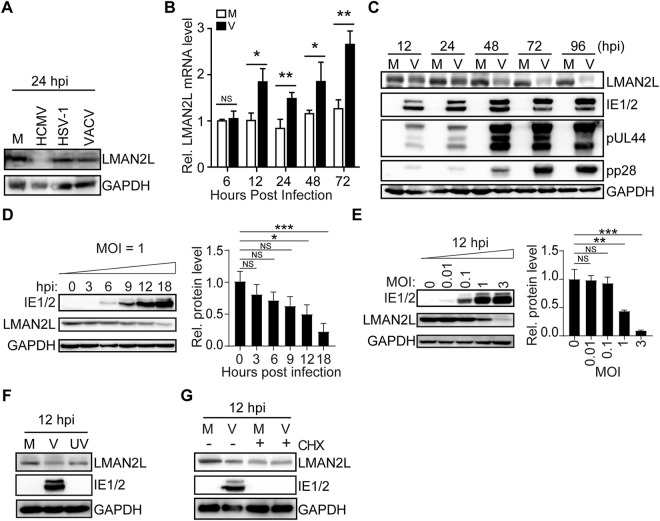
LMAN2L is post-translationally downregulated at the immediate-early stage of HCMV infection. (A) HFFs were infected with HCMV, HSV-1, VACV, or left uninfected. Cell lysates harvested at 24 hours post infection (hpi) were subjected to immunoblotting (IB) for the indicated proteins. (B-C) HFFs were either mock-infected (M) or infected with HCMV AD169 strain (V) at an MOI of 1, and harvested at the indicated time points. LMAN2L mRNA levels (B) were measured by qRT-PCR and normalized to those in mock-infected cells at 6 hpi. LMAN2L and the indicated proteins were analyzed by IB (C). (D-E) HFFs were mock-infected or infected with HCMV (MOI = 1) for 3, 6, 9, 12, or 18 hours (D) or at an MOI of 0.01, 0.1, or 1 for 12 hours (E). LMAN2L and IE1/2 protein levels were analyzed by IB (left panels) and quantified by densitometry, normalized to GAPDH, and presented as relative levels compared to mock-infected controls (right panels). (F) HFFs were mock-infected (M), infected with HCMV (V), or UV-irradiated HCMV (UV) at an MOI of 3. Cell lysates were analyzed by IB for the indicated proteins. (G) HFFs were mock-infected (M) or infected with HCMV (MOI = 1) and treated with DMSO or cycloheximide (CHX; 100 μg/ml) for 12 hpi. Cell lysates were analyzed by IB for the indicated proteins. Statistical significance was determined by one-way ANOVA, with Bonferroni’s post hoc test for multiple comparisons where indicated. *, P < 0.05; **, P < 0.01; ***, P < 0.001; NS, not significant.

We examined LMAN2L expression in human foreskin fibroblasts (HFFs) during infections with various DNA viruses. We found that LMAN2L protein levels were not affected by herpes simplex virus type 1 (HSV-1) and vaccinia virus (VACV), but were reduced by HCMV at 24 hours post infection (hpi; Fig 1A). Next, we assessed how HCMV infection affected LMAN2L transcription and protein levels in HFFs over time. Quantitative RT-PCR (qRT-PCR) revealed that LMAN2L mRNA levels increased significantly at 12 hpi and reached approximately 2.5-fold higher than that in the mock controls by 96 hpi (Fig 1B). However, immunoblot analysis indicated a decrease in LMAN2L protein levels at 12 hpi, which remained low throughout the infection (Fig 1C). Further analysis of the kinetics (Fig 1D) and the effects of varying multiplicities of infection (MOIs; Fig 1E) demonstrated that LMAN2L protein levels decreased in a time- and dose-dependent manner during the immediate-early phase.

Next, we sought to determine whether LMAN2L downregulation depends on newly synthesized viral proteins. HFFs were either inoculated with UV-irradiated HCMV virions to block viral gene expression or treated with the protein synthesis inhibitor cycloheximide (CHX, 100 μg/ml) to block *de novo* protein synthesis. Both UV irradiation and CHX treatment effectively blocked the expression of immediate-early proteins 1 and 2 (IE1/IE2; Fig 1F and 1G). However, neither treatment significantly reduced LMAN2L protein levels compared with the mock group, indicating that *de novo* viral protein synthesis, rather than viral components, is essential for LMAN2L downregulation. Taken together, our findings indicate that the decrease in LMAN2L protein levels during the immediate-early phase is driven by newly synthesized viral proteins.

### HCMV promotes LMAN2L degradation *via* the ubiquitin-proteasome pathway through K48-linked polyubiquitination

**Fig 2:**
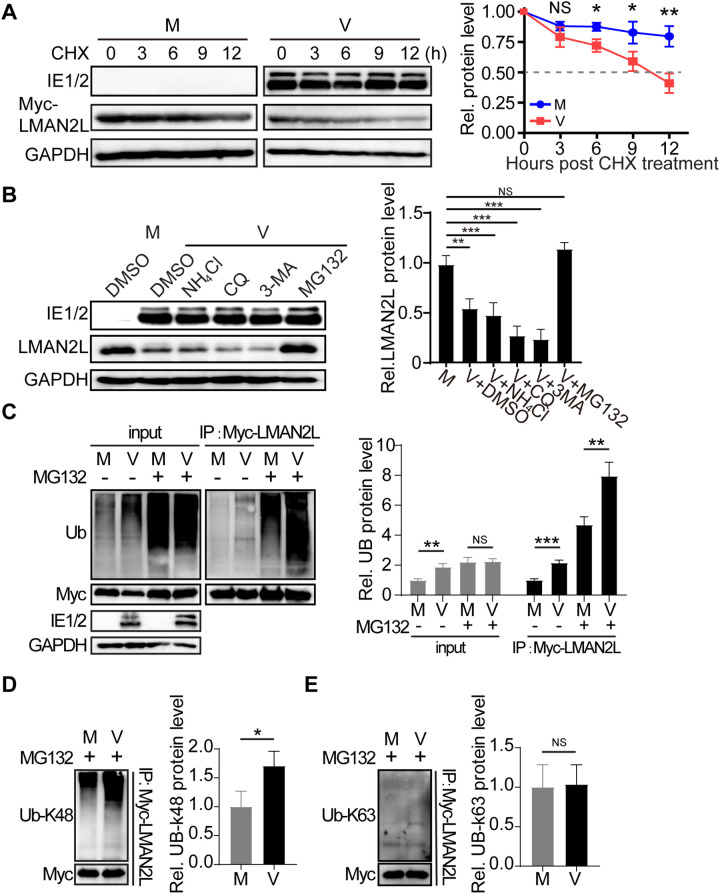
LMAN2L degradation by the ubiquitin-proteasome pathway requires *de novo* viral protein synthesis. HFFs were either mock-infected or infected with HCMV at an MOI of 1. (A) Infected cells were treated with CHX (100 µg/ml) at 0 hpi. LMAN2L protein levels were analyzed by IB at 3-hour intervals post-CHX treatment, normalized to those at 0 hpi (prior to CHX treatment). Representative blots from three independent experiments are shown (left panel); relative protein levels are presented as mean ± SD (right panel). (B) HCMV-infected HFFs were treated with DMSO (vehicle), NH₄Cl (10 mM), chloroquine (CQ; 10 µM), 3-methyladenine (3MA; 10 mM), or MG132 (12.5 µM) at 18 hpi and harvested at 24 hpi. Cell lysates were analyzed by IB for the indicated proteins (left panel). Protein levels were quantified by densitometry, normalized to those in mock-infected cells, and plotted (right panel). (C) HCMV-infected HFFs (overexpressing Myc-LMAN2L) were treated with DMSO (-) or MG132 (12.5 µM; +) at 18 hpi and harvested 6 h post-treatment (24 hpi total). Input lysates were probed by IB with anti-ubiquitin (Ub) or the indicated antibodies (left panel). Myc-LMAN2L was co-immunoprecipitated (co-IP) and IB with anti-Ub antibody (middle panel); ubiquitination levels were quantified by densitometry, normalized to mock-infected controls, and plotted (right panel). (D-E) Myc-tagged LMAN2L immunoprecipitates from HCMV-infected HFFs were analyzed by IB with anti-Ub-K48 (D) or anti-Ub-K63 (E) antibodies (left panels). Protein levels were quantified by densitometry, normalized to those in mock-infected cells, and plotted (right panels). Statistical significance was determined by one-way ANOVA, with Bonferroni’s post hoc test for multiple comparisons where indicated. *, P < 0.05; **, P < 0.01; ***, P < 0.001; NS, not significant.

To dissect the mechanism of HCMV-induced LMAN2L reduction, we first assessed LMAN2L stability in mock- and HCMV-infected HFFs. Cells were treated with CHX to inhibit *de novo* protein synthesis, starting at 12 hpi, and harvested at 3-hour intervals up to 24 hpi. CHX treatment resulted in a much more rapid decline in LMAN2L protein levels in HCMV-infected HFFs compared to mock-infected controls (Fig 2A), suggesting that the infection destabilizes LMAN2L protein.

We then examined the two major pathways of intracellular protein degradation (the autophagosome-lysosome pathway and the ubiquitin-proteasome pathway) using selective inhibitors. As shown in Fig 2B, HCMV infection dramatically reduced LMAN2L levels in DMSO (vehicle)-treated cells. Treatment with the lysosomal inhibitors chloroquine (CQ) and ammonium chloride (NH_4_Cl), or the autophagic inhibitor 3-methyladenine (3MA) had no effect on LMAN2L reduction. In contrast, treatment with the proteasome inhibitor MG132 almost restored LMAN2L levels in infected samples to those of uninfected controls, indicating that HCMV degrades LMAN2L *via* the proteasome route.

These data led us to investigate whether HCMV induces LMAN2L ubiquitination. Due to the lack of commercial LMAN2L antibodies suitable for immunoprecipitation (IP), Myc-tagged LMAN2L was overexpressed in HFFs for the co-immunoprecipitation (co-IP) assays. The cells were infected with HCMV (MOI = 1) for 12 hours and treated with either DMSO or MG132 (12.5 μM). Co-IP was performed with an anti-Myc antibody at 18 hpi, followed by immunoblotting with anti-Ub and anti-Myc antibodies to detect ubiquitinated LMAN2L. As expected, MG132 increased the basal levels of ubiquitinated LMAN2L in mock-infected HFFs (Fig 2C, left panel). In HCMV-infected HFFs, LMAN2L ubiquitination was dramatically enhanced, especially in the presence of MG132 (Fig 2C, right panel). Further analysis of LMAN2L-bound immunoprecipitants with anti-Ub-Lys48 (K48) and anti-Ub-Lys63 (K63) antibodies revealed that HCMV infection promoted the formation of K48-linked polyubiquitin chains on LMAN2L (Fig 2D and 2E). Collectively, these findings suggest that HCMV infection destabilizes LMAN2L by facilitating its ubiquitin-proteasomal degradation through K48-linked polyubiquitination.

### HCMV-pUS2 is indispensable for LMAN2L degradation

**Fig 3:**
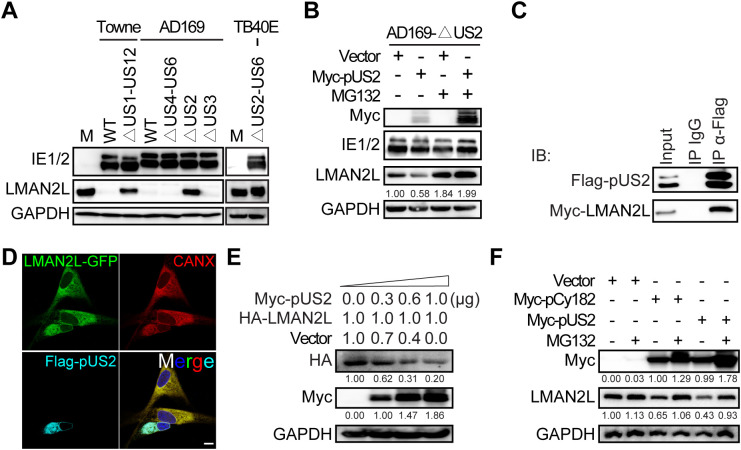
HCMV-pUS2 is essential for LMAN2L degradation *via* the proteasome pathway, which is shared with pCy182 of chimpanzee cytomegalovirus (CCMV). (A) To identify the HCMV gene(s) mediating LMAN2L degradation, HFFs were mock-infected or infected with the indicated HCMV strains and their deletion mutants at an MOI of 1. Cell lysates were harvested at 24 hpi and subjected to IB analysis for the indicated proteins. (B) HFFs stably expressing Myc-tagged pUS2 or empty vector (Vector) were infected with HCMV AD169-ΔUS2 at an MOI of 1. At 24 hpi, cells were ether treated with DMSO (-) or MG132 (12.5 µM; +) for 6 h. Subsequently, cell lysates were subjected to IB analysis for the indicated proteins. LMAN2L protein expression levels were quantified *via* densitometry (values shown below each blot). (C) HEK293T cells were co-transfected with expression plasmids encoding Flag-tagged pUS2 and Myc-tagged LMAN2L for 48 h. Co-IP was performed with anti-Flag antibody, followed by IB analysis with the indicated antibodies. (D) HFFs were co-transfected with plasmids encoding GFP-tagged LMAN2L and Flag-tagged pUS2 for 48 h, then stained with anti-Flag antibody and the ER membrane marker Calnexin (CANX). Nuclei were counterstained with DAPI. Representative confocal microscopy images are shown. Scale bar, 10 μm. (E) HEK293T cells were co-transfected with 1 μg HA-tagged LMAN2L plasmid and different doses of Myc-tagged pUS2 plasmid for 48 h. Vector was added to normalize total plasmid amount in each sample. Cell lysates were subjected to IB analysis for the indicated proteins. Protein levels were quantified *via* densitometry (values shown below each blot). (F) HFFs stably expressing Myc-tagged pUS2, Myc-tagged pCy182 (CCMV orthologue of pUS2), or Vector were treated with DMSO (-) or MG132 (12.5 µM; +) for 6 h. Cell lysates were subjected to IB analysis for the indicated proteins. Protein levels were quantified *via* densitometry (values shown below each blot).

A recent proteomic analysis of chimpanzee cytomegalovirus (CCMV) demonstrated that LMAN2L is among the most significantly upregulated host proteins in cells infected with a CCMV strain lacking the *IRS1–US10* region [34]. This finding implies that the viral factor responsible for LMAN2L downregulation resides within this region. To narrow down potential candidates, we screened HCMV BACs with deletions spanning *US1-US12*. In line with the CCMV study, the Towne-BAC with a deletion in the *US1-US12* region failed to induce the reduction of LMAN2L protein levels, indicating that this region is responsible for LMAN2L degradation (Fig 3A). Since the Towne strain lacks the *UL/b’* region encoding 22 canonical genes, we also used a clinical TB40E BAC that contains this region. Similarly, the TB40E BAC lacking the *US2-US6* region failed to downregulate LMAN2L (Fig 3A), indicating that the *US2-US6* region, rather than the *UL/b’* region, is essential for LMAN2L downregulation. Further examination of this region with AD169 BACs revealed that deletion of *US2*, rather than either *US3* or *US4-US6* regions, significantly restored LMAN2L protein levels. This indicates that pUS2 is critical for LMAN2L degradation. Furthermore, LMAN2L downregulation could be restored in AD169-ΔUS2-infected HFFs by ectopic expression of pUS2, and this restoration remained abrogated by the proteasome inhibitor MG132 (Fig 3B). Our data are consistent with a recent study that identified pUS2 as responsible for HCMV-induced LMAN2L degradation using a distinct yet complementary viral genetic screening strategy [35].

Next, we examined whether pUS2 interacts with LMAN2L. HEK293T cells were co-transfected with Flag-tagged pUS2 and HA-tagged LMAN2L, and IP using Flag antibody confirmed the direct interaction between pUS2 and LMAN2L (Fig 3C). We also analyzed the subcellular localization of pUS2 and LMAN2L in transfected HFFs. Immunofluorescence results revealed that LMAN2L co-localized with pUS2 in the endoplasmic reticulum (ER), and both proteins exhibited robust co-localization with the ER marker CANX (Fig 3D). In addition, co-transfecting HA-tagged LMAN2L with different doses of Myc-tagged pUS2 in HEK293T cells demonstrated that pUS2 downregulated LMAN2L in a dose-dependent manner (Fig 3E).

To determine whether this mechanism is conserved between human and nonhuman primate CMVs, we next examined LMAN2L regulation by HCMV-pUS2 and its CCMV ortholog pCy182. HCMV-pUS2 and CCMV-pCy182 were overexpressed in HFFs using lentiviral vectors and their expression was confirmed by immunobloting (Fig 3F). Overexpression of either pUS2 or pCy182 downregulated LMAN2L compared to the vector control (Fig 3F). These data demonstrates that the mechanism of LMAN2L downregulation is conserved between HCMV and its close relative CCMV, suggesting that pUS2-mediated degradation of LMAN2L represents an evolutionarily conserved immune evasion strategy in primates.

### HCMV-pUS2 directs LMAN2L to ERAD for degradation

HCMV-pUS2 has been shown to direct the major histocompatibility complex class I (MHC-I) to the endoplasmic reticulum–associated degradation (ERAD) pathway for degradation by hijacking the E2 ubiquitin-conjugating enzyme UBE2G2 and the ER-resident E3 ligase RNF139 (also known as TRC8) [36,37]. Given that LMAN2L localizes to the ER [38], we hypothesized and tested whether pUS2 utilizes the same ERAD machinery (i.e., UBE2G2 and RNF139) to promote LMAN2L degradation, as it does for MHC-I.

**Fig 4:**
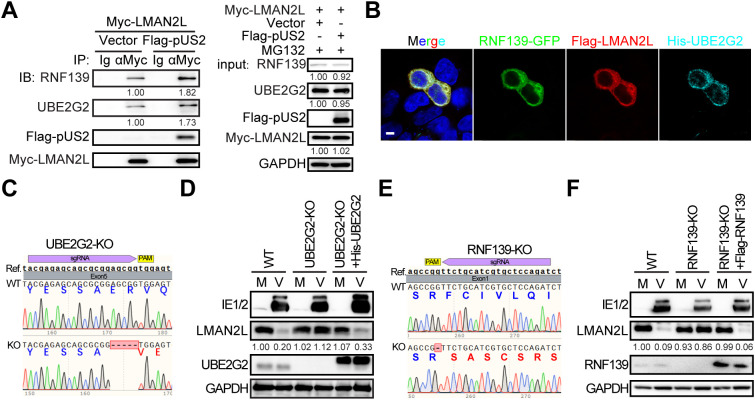
HCMV-pUS2 targets LMAN2L for endoplasmic reticulum–associated degradation (ERAD). (A) HEK293T cells were co-transfected with expression plasmids encoding Myc-tagged LMAN2L and empty vector (Vector) or FLAG-tagged pUS2 for 36 h, and treated with MG132 (12.5 µM) for 6 h. Interactions between ectopic pUS2/LMAN2L and endogenous RNF139/UBE2G2 were detected by co-IP followed by IB analysis with the indicated antibodies. Protein levels were quantified *via* densitometry and normalized to vector-transfected control (values shown below each blot). (B) HEK293T cells were co-transfected with expression plasmids encoding GFP-tagged RNF139, Flag-tagged LMAN2L and His-tagged UBE2G2 for 48 h, and stained with anti-Flag and anti-His antibodies. Nuclei were counterstained with DAPI. Representative confocal microscopy images are shown. Scale bar, 10 μm. (C) To dissect the role of UBE2G2 in pUS2-mediated LMAN2L degradation, UBE2G2 knockout HELFs (UBE2G2-KO) were generated as described in *Materials and Methods*. Genomic DNA was extracted from wild-type (WT) and UBE2G2-KO HELFs, then amplified with UBE2G2-specific primers and analyzed by Sanger sequencing. Single guide RNA (sgRNA) target sequences are highlighted in purple, and PAM motifs in yellow. Representative sequencing chromatograms are shown for WT (top) and UBE2G2-KO (bottom) cells. (D) WT, UBE2G2-KO, and UBE2G2-reconstituted HELFs were infected with HCMV AD169 at an MOI of 1. Cell lysates were harvested at 24 hpi and subjected to IB analysis for the indicated proteins. LMAN2L protein levels were quantified by densitometry, normalized to mock-infected WT HFFs (values shown below each blot). (E) RNF139 knockout HELFs (RNF139-KO) were generated and validated by sequencing as described in (C). (F) WT, RNF139-KO, and RNF139-reconstituted HELFs were infected with HCMV AD169 at an MOI of 1. At 24 hpi, cell lysates were harvested at 24 hpi and analyzed by immunoblotting. LMAN2L levels were quantified by densitometry and normalized to mock-infected WT HFFs (values shown below each blot).

First, we examined whether pUS2, LMAN2L, UBE2G2, and RNF139 assemble into a functional complex in mammalian cells. HEK293T cells were co-transfected with Myc-tagged LMAN2L together with either empty vector (control) or FLAG-tagged pUS2, and then treated with the proteasome inhibitor MG132 (12.5 µM) for 6 h to stabilize protein complexes and block proteasome-dependent degradation. Co-IP was performed using an anti-Myc antibody to enrich LMAN2L-associated complexes. Immunoblotting of the precipitates showed that endogenous RNF139 and UBE2G2 constitutively interacted with LMAN2L under control conditions. Notably, ectopic expression of pUS2 markedly enhanced the association of LMAN2L with both RNF139 and UBE2G2. Meanwhile, FLAG-pUS2 was readily co-precipitated with Myc-LMAN2L, verifying their interaction in cells and indicating that these proteins form a complex (Fig 4A). We further assessed the subcellular localization of LMAN2L, UBE2G2, and RNF139 in transfected HFFs and observed that LMAN2L co-localized with both UBE2G2 and RNF139 at the ER (Fig 4B). Together, these results demonstrate that HCMV-pUS2 functions as a molecular scaffold that reinforces the interaction between LMAN2L and the core ERAD components UBE2G2 and RNF139.

To further determine the roles of UBE2G2 and RNF139 in pUS2-mediated LMAN2L degradation, we established UBE2G2 knockout (UBE2G2-KO) and RNF139 knockout (RNF139-KO) cell lines in hTERT-immortalized HEL cells (HELFs) by CRISPR/Cas9 technology. Successful gene deletions were confirmed by sequencing (Fig 4C and E) and immunoblotting (Fig 4D and 4F). Depletion of UBE2G2 or RNF139 in HCMV-infected cells resulted in a rescue of LMAN2L protein levels (Fig 4D and 4F). Furthermore, reintroducing UBE2G2 into the UBE2G2-KO and RNF139 into RNF139-KO cells restored the HCMV-induced reduction of LMAN2L (Fig 4D and 4F).

Taken together, these results indicate that pUS2 recruits UBE2G2 and RNF139 to form a complex with LMAN2L, thereby redirecting it to the ERAD pathway for ubiquitin-dependent degradation.

### LMAN2L restricts HCMV infection

**Fig 5:**
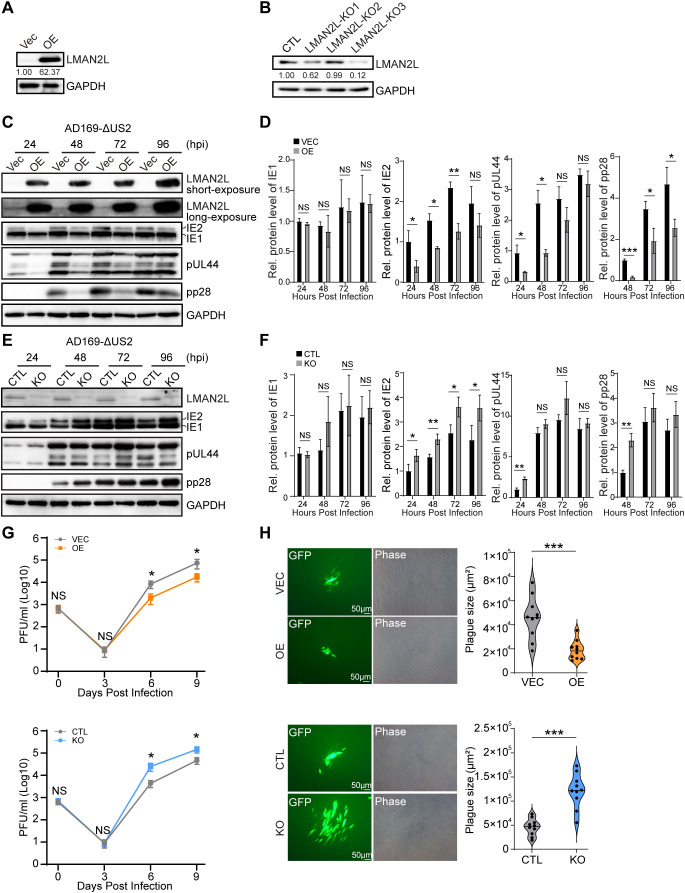
LMAN2L overexpression or knock out modulates HCMV infection. (A and B) To establish stable cell lines, HELFs were transduced with lentiviruses expressing *LMAN2L* cDNA (for overexpression, OE), or three distinct sgRNAs targeting *LMAN2L* (LMAN2L-KO1 to KO3), the corresponding empty vector (Vec for OE, CTL for KO). Transduced cells were selected with puromycin (8 μg/ml) for 3 days. LMAN2L protein levels were assessed by IB and relative protein levels (normalized to CTL or Vec) were quantified and presented below each blot. (C-F) OE, KO, and matched control HELFs were infected with HCMV strain AD169-ΔUS2 at a multiplicity of infection (MOI) of 0.05, and harvested at the indicated time points. (C, E) IB analysis of the indicated viral and cellular proteins, with representative blots from three independent experiments shown. (D, F) Densitometric quantification of IE1, IE2, pUL44 and pp28 protein levels, normalized to GAPDH. IE1, IE2 and pUL44 levels are presented relative to the corresponding infected control at 24 h post-infection (hpi); pp28 levels are normalized to the corresponding infected control at 48 hpi. (G) Viral multi-step growth curves. OE, KO, and matched control HELFs were infected with HCMV AD169-ΔUS2 at an MOI of 0.001. Supernatants were collected at the indicated time points, and viral titers were determined by plaque forming assay. Data were from three independent experiments. (H) Plaque size quantification. Plaque size of AD169-WT and AD169-ΔUS2 in the indicated cells was measured by plaque forming assay as described in the Materials and Methods. Representative plaque images are shown (left panels). The sizes of ten plaques from each group were measured using ImageJ. Data are presented as violin plots, with horizontal lines indicating median values (right panels). Statistical significance was determined by one-way ANOVA. *, P < 0.05; **, P < 0.01; ***, P < 0.001; NS, not significant.

To characterize the role of LMAN2L during HCMV infection, we generated LMAN2L overexpression (OE) and LMAN2L knockout (KO) cell lines. The OE and empty vector control (Vec) cell lines were made by transducing HELFs with a lentivirus expressing the LMAN2L cDNA (GenBank accession No. NM_030805.4) or an empty vector. The LMAN2L-KO and control (CTL) cell lines were generated by transducing HELFs with independent single guide RNAs (sgRNAs) targeting distinct regions of the LMAN2L locus, or a non-targeting control vector. Immunoblotting confirmed robust LMAN2L overexpression in OE cells and efficient knockout in KO clones (Fig 5A and 5B), with the LMAN2L-KO3 clone showing the highest knockout efficiency (Fig 5B) and thus selected for all downstream experiments.

We next investigated the impact of LMAN2L knockout and overexpression on viral protein expression across different kinetic phases. We focused on representative viral proteins from each phase: the immediate-early regulatory proteins IE1 and IE2, the early DNA replication protein pUL44, and the late structural protein pp28. To avoid confounding effects from pUS2-mediated degradation of LMAN2L, all infection assays were performed using the HCMV AD169-ΔUS2 strain at a multiplicity of infection (MOI) of 0.05. Immunoblotting revealed that IE1 protein levels were not significantly altered by either LMAN2L overexpression or knockout across the infection time course (Fig 5C–F). In contrast, IE2 expression was significantly diminished in LMAN2L OE cells relative to Vec controls, and reciprocally upregulated in LMAN2L KO cells compared with CTL controls (Fig 5C–F). For the early protein pUL44, LMAN2L overexpression markedly suppressed its expression during the IE and E phases of infection, with levels normalizing to Vec control levels by the late phase (Fig 5C and 5D). The late viral protein pp28, by contrast, was consistently downregulated in LMAN2L OE cells throughout infection (Fig 5C and 5D). In contrast, LMAN2L KO resulted in significant upregulation of both pUL44 and pp28 at early time points post-infection, with expression levels reached comparable levels to those of CTL controls during the late phase (Fig 5E and 5F).

We next evaluated the impact of LMAN2L modulation on viral replication kinetics using multi-step growth curve analysis. OE, KO, and their matched control cells were infected with AD169-ΔUS2 at a low MOI (0.001), and infectious virus titers in culture supernatants were quantified at serial time points post-infection. As shown in Fig 5G, at 9 days post-infection (dpi), LMAN2L overexpression resulted in ~4.3-fold reduction in infectious virus production relative to Vec controls, whereas LMAN2L knockout led to ~3.1-fold increase in viral titers compared with CTL controls. We further assessed the plaque formation capacity of AD169-ΔUS2 in OE, KO, and their respective control cells. Consistent with the replication kinetics data, at 9 dpi, LMAN2L OE cells displayed ~2.6-fold reduction in plaque size relative to Vec controls, while LMAN2L KO cells showed ~2.4-fold increase in plaque size compared with CTL controls (Fig 5H). Taken together, these data establish that LMAN2L functions as a host restriction factor against HCMV infection.

### LMAN2L depletion attenuates immune response to HCMV infection

**Fig 6:**
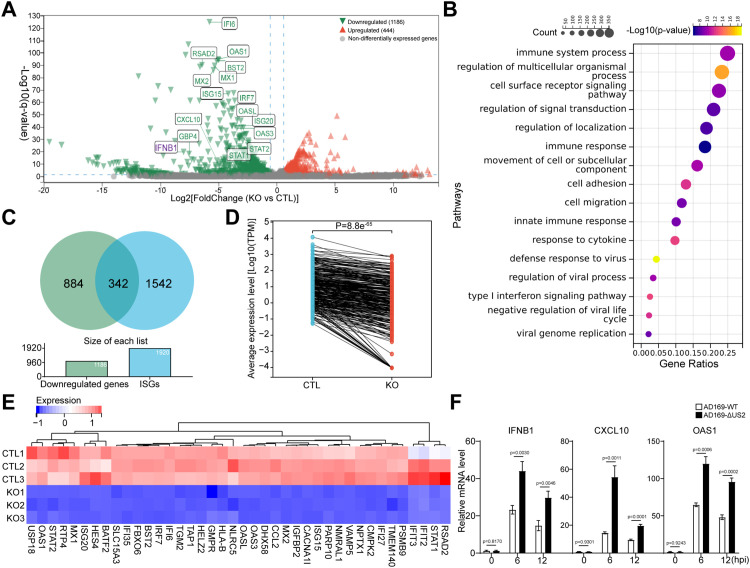
LMAN2L knock out modulates the innate immune response to HCMV infection. (A) LMAN2L-KO (KO) and control (CTL) HELFs were infected with HCMV AD169-ΔUS2 (MOI = 1) for 24 h. RNA-sequencing (RNA-seq) was performed to identify differentially expressed genes (DEGs). Volcano plot showing DEGs between KO and CTL cells [log2 (fold change) vs. -log10 (q-value)]. DEGs with q-value < 0.05 are colored; partial genes with significant differential expression are presented. (B) Top enriched biological process (BP) terms from Gene Ontology (GO) enrichment analysis of DEGs. (C) Venn diagram showing significantly upregulated interferon‑stimulated genes (ISGs) upon HCMV infection. The human ISG gene set was derived from a previous study [71]. (D) Paired line plots illustrating expression levels of the downregulated ISGs in CTL and KO cells. (E) Heatmap showing expression profiles of top 40 interferon-stimulated genes (ISGs). Data represent three biological replicates per group: Control (CTL1–3) and Knockout (KO1–3). Color intensity represents relative expression levels normalized to Z‑scores. (F) HFFs were either mock-infected or infected with AD169-WT or AD169-ΔUS2 (MOI  =  1), collected at the indicated times for expression detection of the indicated antiviral ISGs (*IFNB1, CXCL10, OAS1*) by RT-qPCR. Statistical significance was determined by one-way ANOVA, P-values are shown above the according bars.

A previous study examined the effects of LMAN2L downregulation on cell surface proteins, but its broader influence on host responses during HCMV infection remains unexplored [35]. To assess this gap, we performed RNA-seq analysis during AD169-ΔUS2 infection in the presence or absence of LMAN2L. A comparative analysis of differentially expressed genes (DEGs) between infected control (CTL) and LMAN2L-KO (KO) cells revealed that 1,630 significant DEGs (log2 fold change > 2 and q-value < 0.05), comprising 444 upregulated and 1,186 downregulated genes (Fig 6A and S1 Table). Gene Ontology (GO) analysis of downregulated DEGs highlighted enrichment in immune system processes, cell surface receptor signaling, and cellular localization (Fig 6B), suggesting that LMAN2L may play a role in regulating the innate immune responses to HCMV infection (Fig 6B). Further analysis of the 1,186 downregulated DEGs found 342 of them were ISGs (Fig 6C-D). An analysis of the top 40 most downregulated ISGs identified them as canonical Type I IFN-stimulated ISGs, indicating that the Type I IFN signaling pathway is significantly attenuated in LMAN2L-KO cells (Fig 6E). Since pUS2 promotes LMAN2L degradation, wild-type HCMV should counteract LMAN2L’s antiviral effects by suppressing type I IFN response. To validate this, we infected primary HFFs with AD169-WT or AD169-ΔUS2 and measured representative type I IFN response genes. Compared to AD169-ΔUS2 infection, AD169-WT significantly reduced expression of *IFNB1*, *CXCL10*, and *OAS1*, confirming that pUS2-mediated LMAN2L degradation actively suppresses HCMV-induced type I IFN signaling (Fig 6F).

### LMAN2L modulates antiviral innate immunity through interacting with STING

**Fig 7:**
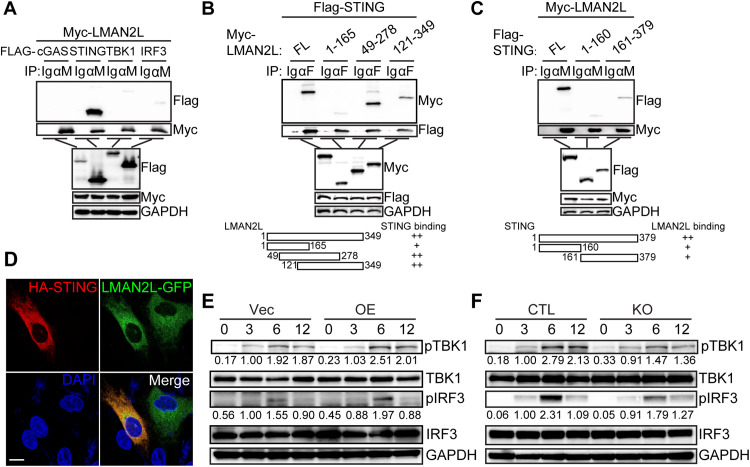
LMAN2L interacts with STING and positively regulates STING pathway activation. (A) To map interactions between LMAN2L and cGAS-STING pathway components, HEK293T cells were co-transfected with Myc-tagged LMAN2L and Flag-tagged cGAS, STING, TBK1, or IRF3 for 24 h. Co-IP was performed with anti-Myc antibody, followed by IB analysis with the indicated antibodies. (B-C) To identify the interaction interface, HEK293T cells were co-transfected with (B) Flag-tagged STING and Myc-tagged LMAN2L truncation mutants, or (C) Myc-tagged LMAN2L and Flag-tagged STING truncation mutants for 24 h. Co-IP was performed with anti-Myc or anti-Flag antibody, followed by IB analysis with the indicated antibodies. (D) HELFs were co-transfected with GFP-tagged LMAN2L and HA-tagged STING for 24 h, then immunostained with anti-HA antibody. Nuclei were counterstained with DAPI. Representative confocal microscopy images are shown. Scale bar, 10 μm. (E-F) To assess the effect of LMAN2L on STING pathway activation, stable LMAN2L-knockout (KO), LMAN2L-overexpression (OE), and their respective control HELFs (CTL for KO, Vec for OE) were infected with HCMV AD169-ΔUS2 (MOI = 1). Cells were harvested at the indicated time points, and cell lysates were analyzed by IB for the indicated proteins. The levels of phosphorylated IRF3 and phosphorylated TBK1 were quantified by gray‑scale scanning using ImageJ. The ratios of phosphorylated to total IRF3 and TBK1 were then normalized to the 6 hpi sample.

Given that the cGAS–STING pathway serves as the primary cytosolic sensor for HCMV to initiate the type I IFN response [11], the observed suppression of type I IFN-stimulated ISGs, despite unchanged transcription levels of core components (*CGAS*, *STING1*, *TBK1*, *IRF3*), prompted us to investigate whether LMAN2L regulates the cGAS-STING signaling axis at the post-translational level. First, we examined the interaction between LMAN2L and key cGAS-STING pathway components. We co-expressed Myc-tagged LMAN2L with Flag-tagged cGAS, STING, TBK1, or IRF3 in HEK293T cells. Co-IP experiments showed that LMAN2L robustly interacted with STING. In contrast, no detectable interaction was observed with cGAS, and interactions with TBK1 and IRF3 were much weaker (Fig 7A). To map the interaction interface between LMAN2L and STING, we performed domain-mapping experiments using truncated constructs of both proteins. We found that the central region of LMAN2L [amino acids (aa) 165–278] was sufficient for binding to STING (Fig 7B). In addition, both the N-terminal (aa 1–160) and C-terminal (aa 161–379) regions of STING were important for binding to LMAN2L (Fig 7C), indicating that LMAN2L interacts with STING at multiple sites. To validate this interaction, we analyzed LMAN2L and STING localization in transfected HFFs and observed their co-localization (Fig 7D).

To further confirm the role of LMAN2L in the STING pathway, we examined the effects of LMAN2L overexpression or deletion on the phosphorylation of TBK1 and IRF3, hallmark markers of STING activation. Our findings revealed that LMAN2L overexpression enhanced the phosphorylation levels of TBK1 and IRF3 in HCMV-infected HELFs, whereas LMAN2L deficiency markedly reduced their phosphorylation level (Fig 7E and 7F). Taken together, these data establish LMAN2L as a positive regulator of the STING pathway, which exerts this function by directly interacting with STING (*via* multiple binding sites) to enhance downstream signaling activation.

### LMAN2L facilitates STING trafficking

STING activation involves its dimerization and trafficking from the ER through the Golgi apparatus to perinuclear microsomes. [15,39]. Once at the Golgi, STING serves as a scaffold to recruit TBK1 and IRF3 to the STING signalosome, ultimately leading to the induction of type I IFNs [40].

**Fig 8:**
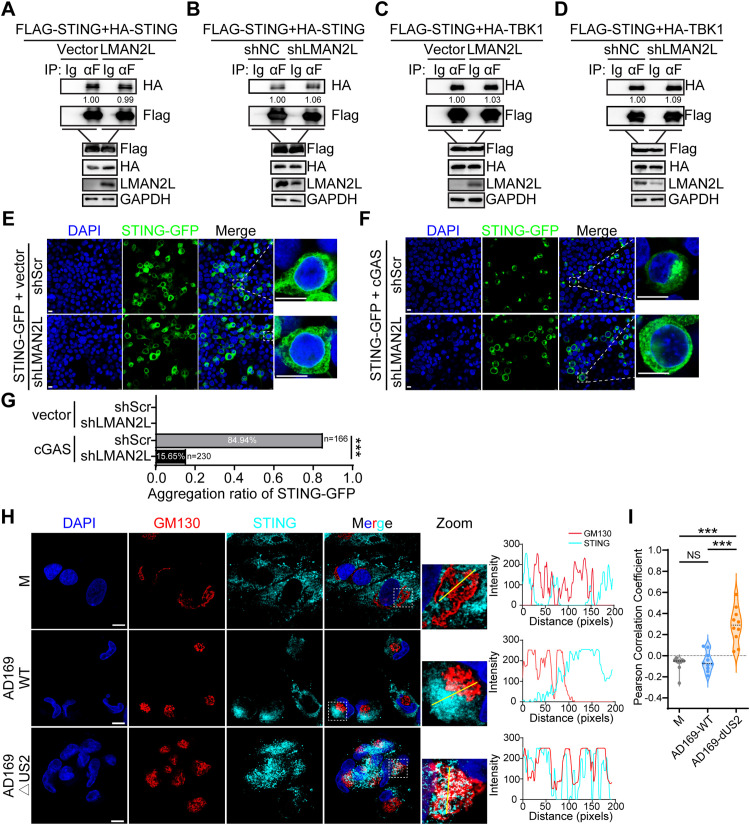
LMAN2L facilitates STING ER-to-Golgi translocation without affecting STING dimerization or STING-TBK1 association. (A-D) HEK293T cells were co-transfected with the indicated plasmids for 24 h. (A-B) Flag-tagged STING and Myc-tagged STING (to assess STING dimerization); (C-D) Flag-tagged STING and Myc-tagged TBK1 (to assess STING-TBK1 association). Co -IP was performed with anti-Flag antibody, followed by IB analysis with the indicated antibodies. (E-F) HEK293T cells were co-transfected with STING-GFP, shLMAN2L (LMAN2L knockdown) or control scrambled shRNA (shScr) for 24 h. Cells were then transfected with Flag-tagged cGAS (+cGAS) or empty vector (+vector) for an additional 6 h to induce STING translocation. Cells were immunostained with anti-Flag antibody; nuclei were counterstained with DAPI. Confocal microscopy images are shown. Scale bar, 10 μm. (G) Quantification of STING perinuclear aggregation ratio from (E-F). Data represent n = 30 counted cells per condition (three independent experiments) and were analyzed using the chi-square test. ***, P < 0.001. (H) HCMV-pUS2 disrupts STING trafficking and translocation to the Golgi apparatus. HFFs were mock-infected or infected with AD169-WT and AD169-ΔUS2. At 24 hpi, cells were harvested and fixed for IFA, counterstained with DAPI and immunolabeled with antibodies against STING (cyan) and GM130 (Golgi marker, red). Images were captured by confocal microscopy. Scale bar, 10 μm. Insets present magnified views of the boxed regions. The fluorescence intensity profile on the right displays signal intensities of the red and cyan channels along the yellow analytical line in the merged images. (I) Co-localization levels and Pearson’s correlation coefficients of STING and Golgi apparatus were analyzed *via* ImageJ software (n = 10). One-way ANOVA followed by Bonferroni post-hoc tests was used for statistical comparison. ***, P < 0.001; NS, not significant.

First, we assessed whether LMAN2L modulates STING dimerization by co-IP. The results demonstrated that neither overexpression (Fig 8A) nor depletion (Fig 8B) of LMAN2L had a significant impact on STING dimerization. We next examined whether LMAN2L is required for STING–TBK1 interaction. The binding affinity of STING to TBK1 remained unaffected in cells with either overexpression (Fig 8C) or depletion (Fig 8D) of LMAN2L. These data demonstrate that LMAN2L does not affect STING-STING and STING-TBK1 interactions.

Since LMAN2L interacts with STING but does not affect its dimerization or binding to TBK1, we next focused on STING trafficking, a key step in STING pathway activation, to determine whether this process is regulated by LMAN2L. We thus detected stimulus-induced STING translocation to perinuclear microsomes following cGAS stimulation. As shown in Fig 8E–G, ectopic cGAS expression strongly drove STING-GFP aggregation at perinuclear regions. Nevertheless, shRNA-mediated LMAN2L depletion significantly impaired this perinuclear STING accumulation with the distribution of STING-GFP resembling that of cells without cGAS stimulation. Together, these data reveal that LMAN2L deficiency blocks stimulus-triggered STING translocation, thereby attenuating downstream STING antiviral signaling cascades.

To further validate STING trafficking regulation during authentic HCMV infection, we examined its subcellular localization in HFFs infected with AD169-WT or AD169-ΔUS2. Mock-infected and AD169-WT-infected cells showed minimal STING-Golgi co-localization. In contrast, strong STING-Golgi co-localization was observed in cells infected by AD169-ΔUS2 (Fig 8H-I). These findings demonstrate that pUS2 restricts STING Golgi trafficking during productive infection.

## Discussion

**Fig 9:**
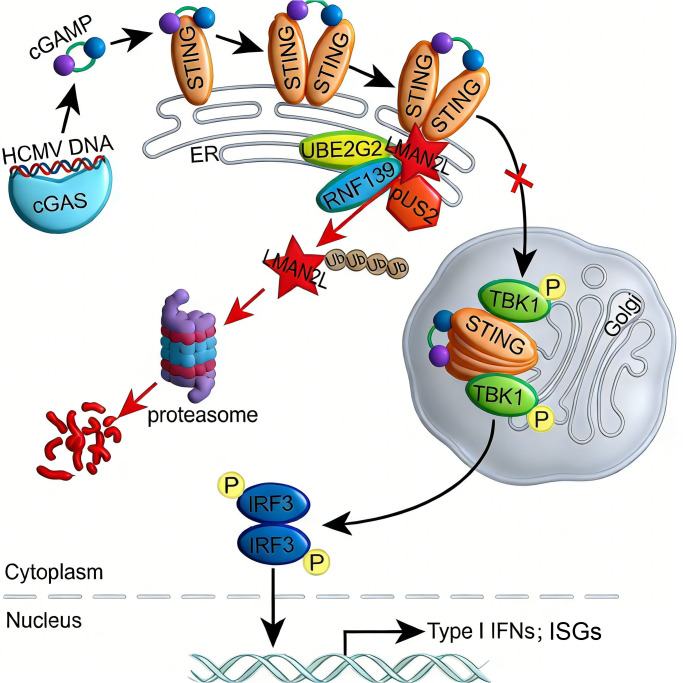
Model of pUS2-mediated HCMV immune evasion by targeting LMAN2L to disrupt STING pathway activation. In response to HCMV DNA, the cytosolic DNA sensor cGAS catalyzes the synthesis of the second messenger cGAMP, which binds to STING. This binding triggers STING dimerization, oligomerization, and subsequent translocation from the ER to the Golgi apparatus. Once at the Golgi, STING recruits TANK-binding kinase 1 (TBK1), which phosphorylates and activates IRF3 to induce type I IFNs and IFN-stimulated genes (ISGs). To evade this innate immune response, HCMV-pUS2 hijacks the host E2 ubiquitin-conjugating enzyme UBE2G2 and E3 ubiquitin ligase RNF139, promotes LMAN2L ubiquitination and proteasomal degradation *via* the ERAD pathway. LMAN2L depletion impairs STING ER-to-Golgi translocation, thereby inhibiting STING-dependent signaling, including TBK1 and IRF3 phosphorylation, as well as IFN-β and ISG induction, ultimately facilitating HCMV immune evasion.

Over millions of years of co-evolution with human hosts, HCMV has evolved sophisticated strategies to evade innate immune surveillance. As a central sensor of cytosolic DNA viruses, the cGAS-STING pathway is targeted and subverted by multiple HCMV-encoded proteins, including pUL31, pUL37 × 1, pUL42, pUL82, pUL83, pUL94, pUL122 (IE2), pUL138 and pUS9 [19,21,23–28,41]. In this study, we identified a new viral protein, pUS2, which targets LMAN2L to antagonize cGAS-STING pathway (Fig 9). We uncover a critical role for LMAN2L in mediating STING ER-to-Golgi translocation during HCMV infection. Additionally, we demonstrate that pUS2 drives LMAN2L degradation through the ERAD pathway, thereby disrupting STING signaling and antiviral defenses. These findings identify LMAN2L as a novel host regulator of STING trafficking and highlight HCMV’s exploitation of ER quality control system as a distinct immune evasion strategy, expanding our understanding of viral-host interactions in innate immunity.

HCMV’s exploitation of host ubiquitin-proteasome systems is not limited to a single strategy. In addition to IE1 as a viral E3 ubiquitin ligase [42,43], HCMV also encodes multiple viral proteins that exploit host E3 ubiquitin ligases to direct host factors for proteasomal degradation. For example, pUL145 recruits the Cullin4-DDB1 E3 ligase complex to facilitate the degradation of TP53 BP1, HLTF and STAT2 [29,44]. Similarly, pUS11 utilizes the E3 ligases Derlin-1 and TMEM129, along with the E2 enzyme UBE2J2, for the proteasomal degradation of MHC-I and the neonatal Fc receptor [45,46]. HCMV-pUS2 was first characterized as targeting MHC-I for rapid proteasomal degradation *via* recruitment of the E3 ligase RNF139 [36,47]. HCMV-pUS2’s substrate repertoire has since expanded to include MHC class II components, cell adhesion molecules (integrins, CD112, thrombomodulin), and signaling receptors (interleukin-12 receptor β1 and PTPRJ) [48,49]. LMAN2L was recently identified as a new target of pUS2 by Weekes’ group [35]. Our findings are consistent with this report; however, our investigation was initiated independently, guided by prior work in chimpanzee CMV implicating the corresponding *IRS1–US10* region [34]. Furthermore, this study performs extensive biochemical analyses to delineate the fine mechanistic details of the E3 ligase-mediated LMAN2L degradation pathway, including the specific polyubiquitin chain linkage driving protein turnover, the cognate E2 ubiquitin-conjugating enzyme required for this process, the assembly of the functional multi-protein degradation complex, and the cross-species evolutionary conservation of this pUS2-mediated degradation event across primate cytomegaloviruses. Together, it deepens our biochemical and functional comprehension of the pUS2–LMAN2L regulatory axis. Regarding the biological consequence of LMAN2L degradation, the previous study examined cell-surface changes linked to the canonical trafficking function of LMAN2L and reported impaired delivery of ITGA6. In contrast, this study revealed broad immune response alterations in the absence of LMAN2L during HCMV infection. Our findings identify LMAN2L as a new regulator of cGAS–STING signaling and expand pUS2’s repertoire beyond the adaptive immune evasion (*via* MHC-I/II targeting) to include the counteraction of innate antiviral responses. This highlights the pleiotropic role of pUS2 in HCMV immune evasion.

STING trafficking through the secretory pathway is critical for innate immune signaling [50]. The ER protein inactive rhomboid protein 2 (iRhom2) recruits the translocon-associated protein β (TRAPβ) to the STING complex to facilitate trafficking of STING from ER to perinuclear microsomes [51]. To date, two HCMV-encoded proteins have been identified to disrupt this STING-iRhom2-TRAPβ translocation complex: pUL82 interacts with iRhom2 to block the STING-TRAPβ interaction, thereby impairing STING trafficking [23], whereas pUL42 acts by directing autophagic degradation of TRAPβ [21]. Emerging evidence suggests that STING exits ER *via* the COPII-dependent ER export pathway [52]. In addition, YIPF5 and STING ER exit protein modulate STING ER exit by facilitating COPII vesicle budding/fusion and stimulating ER membrane curvature, respectively [52,53]. Our findings suggest that the ER-resident lectin LMAN2L also plays a role in the process. LMAN2L binds to high-mannose N-glycans and may serve as a cargo receptor for COPII-coated vesicles, regulating glycoprotein transport to the Golgi [38,54,55]. While LMAN2L has not been directly implicated in COPII vesicle biogenesis, it likely modulates the packaging and transport efficiency of glycoproteins by recognizing specific high-mannose N-glycans. Given that STING undergoes N-glycosylation upon DNA virus infection, a modification critical for its trafficking and immune activation [56], LMAN2L may act as the molecular bridge linking these glycosylated residues on activated STING to the COPII export machinery, thereby facilitating STING ER exit. We also observed weak co-precipitation of TBK1 and IRF3 with LMAN2L (Fig 7A), likely reflecting their indirect recruitment *via* strong binding to activated STING rather than direct LMAN2L interactions. Thus, LMAN2L primarily regulates STING trafficking to initiate cGAS-STING signaling. However, the precise molecular details of how LMAN2L recognizes glycosylated STING and coordinates with the COPII machinery remain unclear, warranting future studies to resolve this interaction and its relevance to STING-mediated innate immunity.

HCMV has evolved a redundant and multi-layered arsenal to subvert the cGAS-STING pathway, deploying distinct viral proteins to target specific nodes for robust immune evasion. Upstream sensing is inhibited by pUL31, which blocks cGAS-DNA binding, and pp65, which prevents the cGAS-STING interaction [19,24]. To deplete the signaling pool, IE2 and pUL138 trigger STING degradation *via* proteasomal and lysosomal pathways, respectively [26,27]. Furthermore, pp71, pUL42, and pUL94 interfere with STING trafficking and oligomerization by targeting the iRhom2-TRAPβ or STING/TBK1 complexes [21,23,25]. In contrast, pUS2 acts indirectly by degrading LMAN2L, the host lectin essential for STING ER-to-Golgi trafficking. This provides additional insurance against STING activation if other mechanisms fail. As an immediate-early protein [57], pUS2 rapidly establishes this blockade, synergizing with later antagonists to disrupt the pathway at multiple temporal and spatial checkpoints. This functional diversity highlights how HCMV maximizes its evolutionary fitness by deploying a coordinated array of inhibitors that collectively dismantle the host antiviral response from sensing to signal transduction.

The antagonism of STING-mediated immunity represents an evolutionarily conserved strategy in the *herpesviridae* family. Both alpha- and gamma-herpesviruses encode diverse proteins that neutralize STING, typically through promoting STING degradation, deubiquitination, or disrupting downstream signaling complexes [58]. Targeting STING ER-to-Golgi trafficking represents a hallmark of beta-herpesviruses, with HCMV deploying multiple proteins (pUL42, pUL82, and pUS2) to block translocation through diverse strategies. Unlike direct antagonists, pUS2 employs a sophisticated approach by recruiting ERAD machinery to degrade LMAN2L, a host facilitator of STING trafficking (Fig 9). While most other herpesviruses lack such mechanisms, HSV-1 γ134.5 impacts this step through an incompletely defined mechanism [59]. The absence of US2 homologs in alpha- and gamma-herpesviruses, combined with our observation that HSV-1 and VACV fail to downregulate LMAN2L, establishes this mechanism as a unique *betaherpesvirinae* adaptation. This specialized reliance on host ER quality control likely reflects CMV’s distinct co-evolutionary path.

In summary, this study identifies LMAN2L as a key regulator of STING trafficking and reveals that HCMV-pUS2 disrupts STING signaling by exploiting ERAD to degrade LMAN2L (Fig 9). These findings expand pUS2’s role from suppressing adaptive immunity to antagonizing STING signaling, and suggest LMAN2L as a potential therapeutic target for viral infections, and STING-associated inflammatory diseases.

## Materials and methods

### Ethics statement

Human foreskin fibroblasts (HFFs) were isolated from neonatal human foreskins and have been preserved in our laboratory for years. The cell isolation protocol was approved by the Institutional Review Board (IRB) of the Wuhan Institute of Virology, Chinese Academy of Sciences (approval number: WIVH10201202), in accordance with the Guidelines for Biomedical Research Involving Human Subjects [60]. Additionally, the tissue was obtained from a postmortem fetus and written informed consent was waived.

### Cells and Regents

HFFs were isolated and maintained as described previously [60]. Human embryonic kidney 293T (HEK293T) cells were purchased from ATCC (CRL-11268) and cultured in Dulbecco’s modified Eagle’s medium (DMEM; cat. no. 41500–034; Thermo Fisher Scientific) supplemented with 10% fetal bovine serum (FBS; cat. no. SXRS-FBS-001; RUNSUN Biotech) and penicillin-streptomycin (100 U/ml and 100 μg/ml, respectively; cat. no. 15140–122; Thermo Fisher Scientific). The human embryonic lung fibroblast (HELF) cell line immortalized with human telomerase reverse transcriptase (hTERT) was kindly provided by Jason J. Chen (Guangzhou Medical University) [61].

Both HFFs and HELFs were cultured in minimum essential medium (MEM; cat. no. 41500–034; Thermo Fisher Scientific) supplemented with 10% FBS and penicillin-streptomycin as described above. All cells were incubated at 37°C in a humidified atmosphere containing 5% CO₂.

The following reagents and antibodies were purchased from the indicated manufacturers: MG132 (cat. no. HY-13259; MCE), chloroquine (cat. no. HY-17589A; MCE), 3-methyladenine (cat. no. HY-19312; MCE), ammonium chloride (cat. no. HY-Y1269C; MCE), puromycin dihydrochloride (cat. no. HY-B1743A; MCE), and G418 selective antibiotic (cat. no. HY-K1056; MCE). Mouse monoclonal antibodies against IE1/2 (cat. no. P1215; Virusys), pUL44 (cat. no. P1202-1; Virusys), pp28 (cat. no. CA004-1; Virusys), Myc (cat. no. 60003–2-Ig; Proteintech), and Flag (cat. no. F3165; Merck) were used. Rabbit polyclonal antibodies against Myc (cat. no. 16286–1-AP; Proteintech), Flag (cat. no. 20543–1-AP; Proteintech), HA (cat. no. 51064–2-AP; Proteintech), LMAN2L (cat. no. A18505; Abclonal and cat. no. 17877–1-AP; Proteintech), GM130 (cat. no. 610822; BD Transduction Laboratories); ubiquitin (cat. no. A0162; Abclonal), K48-linkage-specific ubiquitin (cat. no. A3606; Abclonal), K63-linkage-specific ubiquitin (cat. no. A18164; Abclonal), UBE2G2 (cat. no. A10408; Abclonal), STING (cat. no. A21051; Abclonal), STING (cat. no. 66680–1-Ig; Proteintech); TBK1 (cat. no. 28397–1-AP; Proteintech), IRF3 (cat. no. 11312–1-AP; Proteintech), phosphorylated (p)-TBK1 (S172; cat. no. ab109272; Abcam), p-IRF3 (S386; cat. no. ab76493; Abcam), and GAPDH (cat. no. 10494–1-AP; Proteintech) were also used.

### Viruses and infections

Virus stocks were produced and titrated *in vitro* using standard techniques [33,62,63]. HSV-1 H129 strain BAC was generated as described previously [64]. VACV Western Reserve Strain was kindly provided by Yun Wang (Wuhan Institute of Virology, CAS) [65]. HCMV Towne strain (ATCC VR-977) was purchased from ATCC. The Towne-BAC strain, which lacks the entire *US1-US11* region and truncates *IRS1* and *US12*, was kindly provided by Hua Zhu (Rutgers University) [66]. Wild-type HCMV AD169 BAC, AD169-ΔUS4-US6 BAC, and TB40E-ΔUS4-US6 BAC were kindly provided by Zhi-Kang Qian (Institute Pasteur of Shanghai, CAS). The HCMV AD169-BAC strain (originally designated pAD/Cre) contains a self-excisable BAC cassette inserted between *US28* and *US29* [67]. The AD169-ΔUS4-US6 strain (originally designated pAD-GFP) was generated by replacing the *US4-US6* region of pAD/Cre with a GFP expression cassette [68]. The clinical TB40E-ΔUS4-US6 BAC (originally designated TB40-BAC4) has a BAC cassette inserted between *US2* and *US6* [69].

To generate AD169-ΔUS2 and AD169-ΔUS3 BACs, the *US2* and *US3* coding sequences were deleted *via* ampicillin resistance (Ampᵣ) selection. Briefly, the *US2* and *US3* regions were replaced with an Amp^R^-SV40 promoter-GFP cassette by homologous recombination between AD-Cre BAC and PCR-amplified fragments. The primers used were as follows: Forward-dUS2: 5′-CTCTGGGATATAAATTGGGAAAGAGCGTACAGTCCACACGCTGTTTCACCTTACCAATGCTTAATCAGTGAGG-3′; Reverse-dUS2: 5′-AGATCGTGACCATTATCACCAAGATAGTTCCCACCATAATTCCCATCGTCACTAGAGTCGGACCATGATTACGCCAAGCTCC-3′; Forward-dUS3: 5′-GCAGCCAGACCGGAGCGGTGAGCGGAGCCGAGCAGCGGACCTTCGGAGCCTTACCAATGCTTAATCAGTGAGG-3′; Reverse-dUS3: 5′-CCGTACCTTGCAGCCCAGGTAGGTTTCAGGTACCAGCTGGTTCGTACCTGGCAGGCGGCCGCTTTACTTGTAC-3′. pAD-GFP and pUC19 were used as templates for PCR amplification.

### Plaque formation and size assays

Human foreskin fibroblasts (HFFs) were seeded at a density of 2.5 × 10^6^ cells per well in 24-well plates. Upon reaching confluence, the cells were rendered quiescent. They were then infected with the indicated virus at a multiplicity of infection (MOI) of 0.001 for 3 h, followed by the application of MEM with a 0.5% agarose overlay. Plaques were allowed to develop for 8 days prior to analysis. Individual plaques were identified as distinct foci of GFP-positive cells exhibiting cytopathic effects (CPE). Plaque sizes were quantified using ImageJ software.

### Quantitation of virus replication

Virus multi-step growth curves were determined as previously described [61]. Cells were infected with AD169-WT or AD169-ΔUS2 at a low MOI of 0.001 to allow for multiple rounds of replication, and supernatants were collected at indicated time points and stored at -80°C until they were assayed. Following 10-fold serial dilution, infectious titers were quantified *via* plaque assay as described above.

### Co-immunoprecipitation (Co-IP) and immunoblotting (IB)

HEK293T cells or HFFs were lysed in cell lysis buffer (cat. no. P0013; Beyotime) containing a protease inhibitor cocktail (cat. no. 04693159001; Roche). Protein concentrations of cell lysates were determined using the Bradford assay (cat. no. 500–0205; Bio-Rad). For each immunoprecipitation, 1 mg of lysate was incubated with control IgG or the indicated antibody (1 μg) at 4°C overnight with gentle rotation, followed by incubation with 30 μl of Protein A + G-agarose beads (cat. no. P2012; Beyotime) at 4°C for 3 h. Beads were collected by centrifugation and washed five times with 1 ml of cell lysis buffer. Immunoblotting was performed as described previously [33,62,63].

### Quantitative reverse transcription-PCR (qRT-PCR)

Cells were infected at an MOI of 1 and harvested at the indicated time points post-infection. Total RNA was extracted from 5 × 10⁵ cells using RNAiso Plus reagent (cat. no. 9109; TaKaRa) and reverse-transcribed into cDNA. Real-time PCR was performed as described previously [33,62,63]. The mRNA levels of target genes were normalized to that of human *GAPDH*. Gene-specific primer sequences are as follows: *LMAN2L*, Forward: 5′-TGTGGGGCTGGGAGTATTTG-3′ and Reverse: 5′-CATAGCTGAGGGAGCCGTTG-3′; *GAPDH*, Forward: 5′-GAGTCAACGGATTTGGTCGT-3′ and Reverse: 5′-GACAAGCTTCCCGTTCTCAG-3′; *IFNB1*, Forward: 5′-AGGACAGGATGAACTTTGAC-3′ and Reverse: 5′-TGATAGACATTAGCCAGGAG-3′; *CXCL10*, Forward: 5′-GGTGAGAAGAGATGTCTGAATCC-3′ and Reverse: 5′-GTCCATCCTTGGAAGCACTGCA-3′; *OAS1*, Forward: 5′- GTCTCCAAGAAGGGGGACCT-3′ and Reverse: 5′- TTGCATCAGTGCCATCTCTGT-3′.

### Plasmids construction and lentiviruses preparation

The human *LMAN2L*-coding sequence (GenBank accession No. NM_015358.3), *RNF139*-coding sequence (GenBank accession No. NM_007218.4), and *UBE2G2*-coding sequence (GenBank accession No. NM_003343.6) were amplified from total RNA of HFFs by reverse transcription-PCR (RT-PCR). The *US2* gene was amplified from the AD169 BAC genome (GenBank accession No. FJ527563.1). The CCMV-*Cy182* gene (from BAC-Phan9; GenBank accession No. MZ151943.1) was synthesized by Tsingke Biotechnology. Mammalian expression plasmids encoding Flag-, Myc-, His-, or HA-tagged versions of these genes and their truncation mutants were constructed using standard molecular cloning techniques. Expression plasmids for HA-tagged ubiquitin, Flag-tagged cGAS/STING/TBK1/IRF3, and HA-tagged cGAS/STING/TBK1/IRF3 have been described previously [61,70].

LMAN2L shRNAs (sh1: 5′-UUUGGAAACAUGGACAAAUUU-3′; sh2: 5′-CAAACGUUCGAGUACUUGAAA-3′; sh3: 5′-GAAUCUGCAUGGGGAUGGCUU-3′) and control scrambled shRNA (shScr: 5′-UUCUCCGAACGUGUCACGU-3′) were inserted into the pCDSHR vector [33]. Single-guide RNAs (sgRNAs) targeting *LMAN2L*, *RNF139*, and *UBE2G2* were designed using the GPP sgRNA Designer tool (https://portals.broadinstitute.org/gpp/public/analysis-tools/sgrna-design) The sgRNA sequences were as follows: *LMAN2L* (sgRNA1: 5′-GCGGCGATGTTTGTCGGCTC-3′; sgRNA2: 5′-CTGGGGTAAGGCGGATATAC-3′; sgRNA3: 5′-TGTCGGCTCGGGATGGGTCC-3′), *RNF139* (5′-TCTGGAGCACGATGCAGAAC-3′), and *UBE2G2* (5′-ACGAGAGCAGCGCGGAGCGG-3′). These sgRNAs were cloned into the BbsI site of the lentiviral vector pLentiCRISPR-E (Addgene; cat. no. 78852). Lentiviruses were prepared by co-transfecting the sgRNA-expressing construct, psPAX2 (packaging vector), and pMD2.G (envelope vector) into HEK293T cells, as described previously [33,61].

### Lentiviral transduction of HELF cells

To generate HELF cells stably overexpressing *LMAN2L*, HELFs were infected with *LMAN2L*-expressing lentiviruses. Infected cells were selected with 8 μg/ml puromycin for 3 days. A similar approach was used to generate *LMAN2L*-, *RNF139*-, or *UBE2G2*-knockout HELFs *via* lentiviral transduction of sgRNA-expressing constructs. Cell colonies were isolated by flow cytometry sorting, seeded into 96-well plates, and screened for the loss of target protein expression by IB. Genomic DNA was extracted from positive clones and subjected to Sanger sequencing for validation.

### Immunofluorescence analysis (IFA)

Synchronized HELF-derived cells or HEK293T cells were seeded onto glass coverslips. Cells were fixed with 4% paraformaldehyde, and target proteins were detected by incubation with primary antibodies followed by appropriate fluorescently labeled secondary antibodies, as described previously [33,61]. Nuclei were counterstained with DAPI (cat. no. D9542; Sigma-Aldrich). Images were acquired using Andor software (PerkinElmer) on a PerkinElmer UltraVIEW VoX spinning-disk laser confocal scanning microscope.

### RNA sequencing (RNA-Seq) and bioinformatics

Three biological replicates of LMAN2L-KO and CTL HELFs were infected with HCMV AD169-ΔUS2 at a multiplicity of infection (MOI) of 1 for 24 h. RNA-seq was performed by Sangon Biotech Co., Ltd. (Shanghai, China). Total RNA was extracted using TRIzol reagent, and individual cDNA libraries were constructed for each replicate. Sequencing was conducted to generate 150-bp paired-end reads. Clean reads were aligned to the human reference genome (hg38) using HISAT2 (version 2.0) with default parameters. Gene expression levels were quantified as transcripts per million (TPM). Differentially expressed genes (DEGs) were identified using DESeq2 (version 1.12.4) with thresholds of a q-value < 0.05 and an absolute log2 fold change > 1.5. Functional enrichment analyses, including Gene Ontology (GO) terms, were subsequently performed.

### Statistical analysis

Data were collected from three independent experiments and analyzed using SPSS software (version 18.0; SPSS) with chi-square tests or one-way analysis of variance (ANOVA), as appropriate. For multiple group comparisons, Bonferroni’s post hoc test was performed following one-way ANOVA to correct for multiple testing. Results are presented as mean ± standard deviation (SD) or median ± SD. A P-value < 0.05 was considered statistically significant.

## Supporting information

S1 Table Raw RNA-seq datasets and GO functional enrichment analysis of 1630 differentially expressed genes in AD169-ΔUS2-infected cells with or without LMAN2L expression (related to S1 Fig). (XLSX)
